# Type 2 diabetes mellitus exacerbates vaginal group B *Streptococcus* colonization via impaired mucosal cytokine response

**DOI:** 10.1128/msphere.00027-26

**Published:** 2026-07-10

**Authors:** Clare M. Robertson, Vicki Mercado-Evans, Addison B. Larson, Holly Branthoover, Samantha Ottinger, Marlyd E. Mejia, Zainab A. Hameed, Lindsey A. Gonzalez, Camille Serchejian, Libbie Ogilvie, Jacob J. Zulk, Kathryn A. Patras

**Affiliations:** 1 Department of Molecular Virology and Microbiology, Baylor College of Medicine 189531, Houston, Texas, USA; 2 Alkek Center for Metagenomics and Microbiome Research, Baylor College of Medicine 661296, Houston, Texas, USA; University of Michigan, Ann Arbor, Michigan, USA

**Keywords:** diabetes, vaginal microbiota, vaginal mucosal immunity, group B *Streptococcus*

## Abstract

Type 2 diabetes mellitus (T2D) is a metabolic disorder that confers increased risk of microbial infections, including those caused by the opportunistic pathogen group B *Streptococcus* (GBS). Asymptomatic GBS vaginal carriage is a notable reservoir for infection, but the impact of T2D on the vaginal mucosa and GBS colonization is not fully understood. We employed a diet-induced mouse model of T2D to investigate the impact of diabetes on glucose availability, vaginal microbiome composition, and vaginal cytokines at baseline and in response to GBS. We observed enhanced susceptibility of diabetic mice to GBS vaginal colonization and reproductive tract dissemination. Despite experiencing hyperglycemia, diabetic mice did not exhibit elevated glucose in the reproductive tract. Regarding the vaginal microbiota, diabetic mice had minimal compositional differences, with decreased *Mammaliicoccus* being the only significant taxonomic variance. Vaginal cytokine profiling revealed consistently depressed cytokines in diabetic mice, beginning with KC at baseline and expanding to eight pro-inflammatory cytokines post-GBS infection. Diabetic mice exhibited decreased proportions of uterine neutrophils and, following GBS exposure, also displayed an expanded vaginal γδ T cell compartment compared with controls. Pairing cytokine observations with GBS colonization revealed a correlation between delayed vaginal IL-1α induction and persistent vaginal GBS, suggesting that vaginal cytokine deficiency may contribute to diabetic GBS phenotypes. Intravaginal supplementation with rIL-1α resolved GBS burden differences between diabetic mice and controls, confirming that deficient vaginal cytokines contribute to diabetic GBS vaginal persistence. These findings advance our understanding of diabetic vaginal mucosal susceptibility to pathogens and support the potential for immunological intervention.

**IMPORTANCE:**

People with T2D are more susceptible to microbial infections, but there is limited understanding of the mechanisms that drive this vulnerability. One possibility is that T2D enhances the colonization of opportunistic pathogens, like GBS, in mucosal reservoirs as a precursor to infection. In this study, we used a diabetic mouse model to test whether diabetes alters the vaginal mucosa to promote GBS colonization. We found that increased vaginal GBS colonization in diabetic mice was not linked to tissue glucose availability or changes in the vaginal microbiome but instead was associated with impaired vaginal immune responses. These findings provide a foundation for translational approaches to reduce GBS persistence and dissemination in at-risk individuals.

## INTRODUCTION

Group B *Streptococcus* (GBS; *Streptococcus agalactiae*) is a gram-positive bacterium that asymptomatically colonizes the gastrointestinal and urogenital tracts of approximately one in five non-pregnant adults (1). While healthy, non-pregnant adults are generally impervious to invasive infection; those with metabolic disorders are more susceptible to a range of manifestations, including GBS, bacteremia, and skin and soft tissue infections (2, 3). In two U.S.-based bacterial surveillance studies, diabetes mellitus and/or obesity were the most common underlying conditions found in GBS non-pregnant adult invasive infections (4, 5). Diabetes mellitus is a metabolic disorder that can be classified into three main types: type 1 (destruction of beta-cells, insulin-dependent), type 2 (imbalance between insulin production and sensitivity), and gestational (manifests in pregnancy), with type 2 (T2D) being the most common (90% of diabetic cases), impacting more than half a billion people worldwide (6–8). In pregnancy, gestational diabetes elevates the risk of rectovaginal GBS colonization by 16%, whereas pregestational diabetes (either type 1 or type 2) elevates the risk by 76% (9). Although maternal GBS colonization is a well-recognized risk factor for invasive disease in the neonatal period (10, 11), the unique diabetic susceptibility to GBS colonization outside of pregnancy, including the vaginal reservoir, is underexplored. While some, but not all, clinical studies suggest a male bias for GBS invasive disease in non-pregnant adults (5, 12, 13), a vaginal GBS reservoir may serve a uniquely important role in diseases disproportionately impacting women, including urinary tract infection and vaginal disorders such as aerobic vaginitis (14, 15).

Multiple characteristics of T2D could explain susceptibility to GBS vaginal colonization. Three major contributors to susceptibility to pathogens during diabetes are thought to be defective glucose homeostasis, altered composition of the resident microbiota, and aberrant immune recognition and response to pathogens (16–19). Hyperglycemia is thought to increase glucose availability at the mucosal sites where the microbiota resides, which could improve pathogen fitness through direct consumption or modulation of virulence factor expression. Clinically, poor glycemic control is positively associated with GBS colonization risk in women with pregestational diabetes (20); however, a pubertal-onset obesity murine model found no correlations between GBS vaginal burden and systemic glucose intolerance (21). *In vitro* studies have demonstrated widespread transcriptional changes in GBS in response to elevated glucose, including genes related to virulence and host cell adherence (22, 23). Studies that measure vaginal glucose during diabetes or hyperglycemia are sparse and present conflicting results (24–26); thus, it remains unclear whether T2D alters vaginal glucose availability and, in turn, GBS colonization fitness.

The vaginal microbiota is a community of microorganisms that, in a healthy state, exerts control over pathogen invasion and survival via direct competition or indirect modulation of host responses (27). GBS colonization correlates with the absence of beneficial *Lactobacillus* spp. and presence of non-optimal vaginal taxa in both non-pregnant (28, 29) and pregnant (30–33) populations, and many of these synergistic and antagonistic relationships have been established experimentally (34–36). Although taxonomically distinct from the human vaginal microbiota, murine models have also identified correlations between vaginal community composition and GBS colonization success in non-pregnant models (36–38) and GBS infection outcomes in healthy and gestational diabetic pregnancy (39). However, the impact of metabolic disease on the vaginal microbiota is not well-understood. Animal models of pubertal-onset obesity (21) and gestational diabetes (39) have reported modest alterations to vaginal bacterial composition. In humans, vaginal microbial composition in T2D is an active area of research, with recent studies indicating a shift to a higher risk infection-associated *Lactobacillus* spp. replete profiles (40–43), but the extent to which these alterations influence pathogen colonization is uncharacterized.

Upon vaginal colonization, GBS induces innate immune cytokine and chemokine production by the human vaginal epithelium *in vitro* (such as IL-8 and IL-1β) (44–46) and mice *in vivo* (such as KC, IL-1β, and IL-17) (39, 45), and subsequent cellular immune responses, particularly neutrophils and γδ T cells, are critical in reducing GBS vaginal burdens (39, 47). Diabetes is widely appreciated to coincide with chronic low-grade systemic inflammation, which may be counterproductive for effective pathogen responses (48, 49). Indeed, neutrophils from T2D patients display reduced GBS phagocytosis in experimental hyperglycemia (50). In the context of urogenital immune responses to GBS, diabetic phenotypes appear context-dependent. In response to GBS UTI, type 1 diabetic mice display elevated immune cell recruitment (51), whereas type 2 diabetic mice have reduced immune cell recruitment (52). In models of gestational diabetes, experimental hyperglycemia in human placental explants reduced cytokine production in response to GBS infection (53), whereas diabetic mice produced exacerbated KC and G-CSF levels when colonized with GBS (39). Whether vaginal immune responses to GBS are altered in T2D has not been established.

Given this site’s potential to serve as a reservoir for GBS to disseminate to other body sites and cause invasive infection, the present study aims to address the role of metabolic parameters, the vaginal microbiota, and mucosal immunity in mediating increased T2D susceptibility to GBS vaginal colonization. Using a diet-induced T2D model in reproductive-age mice, we performed integrated analyses of metabolic phenotypes such as glucose intolerance and vaginal glucose availability, vaginal microbiome profiling, and vaginal cytokine quantification with GBS colonization outcomes. These findings advance our current understanding of the impact of T2D on the vaginal environment and support the potential for immunological intervention to control GBS vaginal colonization in this susceptible population.

## RESULTS

### A high-fat, high-sucrose diet causes aberrant metabolism and exacerbates GBS vaginal colonization and dissemination

**Fig 1:**
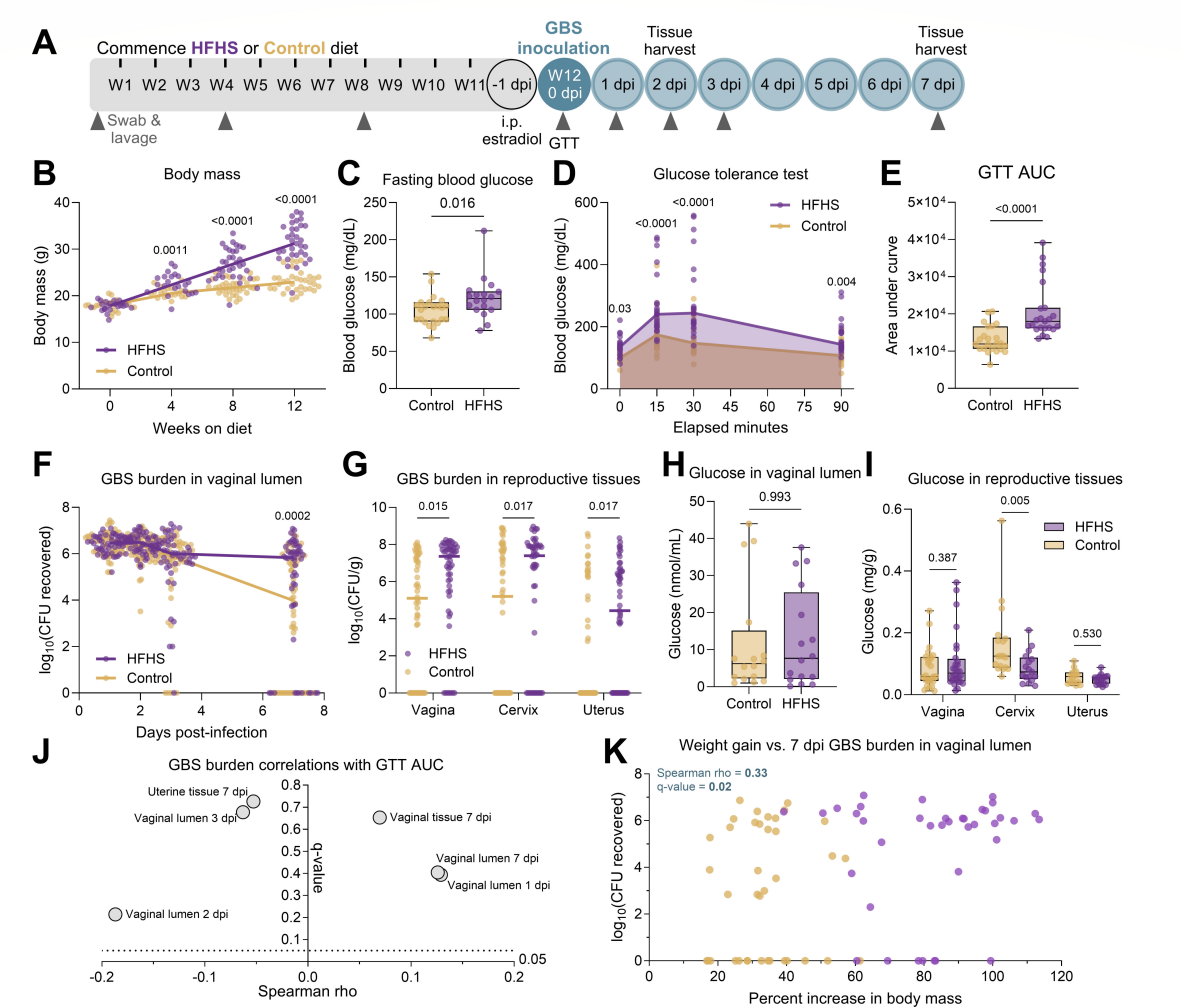
HFHS diet-induced type 2 diabetes exacerbates group B *Streptococcus* vaginal colonization and reproductive tract ascension. (**A**) Experimental timeline of type 2 diabetes development on 12 weeks (W) of high-fat, high-sucrose (HFHS) and control diets, and subsequent GBS CNCTC 10/84 vaginal colonization and sample collection. (**B**) Body mass over time. (**C**) Fasting blood glucose at 12 weeks on diet. (**D**) Blood glucose concentrations during the glucose tolerance test. (**E**) Area under the curve (AUC) from the glucose tolerance test (GTT). (**F**) GBS burdens from post-infection vaginal swabs. (**G**) GBS burdens from homogenized reproductive tissues at 7 dpi. (**H**) Glucose concentrations in vaginal lavage fluid after 12 weeks on diet. (**I**) Glucose concentrations in post-infection reproductive tissues. (**J**) Spearman correlations between GBS swab and tissue burdens and GTT AUC. (**K**) Spearman correlation between body mass increase and 7 dpi vaginal swab GBS burdens. *n* = 18–34 (**B**), *n* = 18–23 (**C–E, J**), *n* = 53–54 (**F, G**), *n* = 16 (**H**), *n* = 16–28 (**I**), and *n* = 76 (**K**). Data represent 4 (**B–E, I–K**), 3 (**H**), and 8 (**F, G**) independent experiments. Samples were assayed in duplicate (**H, I**). Points indicate individual samples, and lines or curves indicate medians. Box and whisker plots show all points and extend from minimum to maximum (**C, E, H**). Data were analyzed by two-way ANOVA with Benjamini, Krieger, and Yekutieli correction, and false discovery rate (FDR) was set at 5% (**B, D, F, G, I**), two-tailed Mann−Whitney *U* test (**C, E, H**), and Spearman correlation with Benjamini-Hochberg FDR correction (**J, K**).

To assess the impact of diabetes on susceptibility to GBS, we paired murine models of diet-induced T2D and GBS vaginal colonization (54, 55). Adult C57BL/6J female mice were provided a high-fat, high-sucrose (HFHS, 45% kcal from fat) diet or a low-fat, no sucrose (control, 10% kcal from fat) diet for 12 weeks. Every 4 weeks, body mass, vaginal swabs, and vaginal lavage were collected, and a glucose tolerance test was performed at 12 weeks as indicated in Fig. 1A. Compared to controls, HFHS-fed mice displayed significantly greater body mass gain as early as 4 weeks after starting diet (Fig. 1B), and fasting blood glucose was significantly elevated by 8 weeks on diet (Fig. 1C). HFHS-fed mice had impaired glucose tolerance as indicated by heightened blood glucose following glucose bolus and an increased area under the curve compared to control mice (Fig. 1D and E).

After 12 weeks on a diet, mice were vaginally inoculated with GBS strain CNCTC 10/84 (serotype V) or A909 (serotype Ia), representing two of the most common serotypes infecting both diabetic and non-diabetic populations (56–58). CNCTC 10/84 has a mutation in the *covR* promoter (59), the regulatory component of the covRS system that coordinates GBS virulence and response to glucose (22). However, in the context of vaginal infection, CNCTC 10/84 displays lower colonization persistence compared to other strains, including the serotype V strain CJB111, which shows persistent colonization for a month or more (55). Thus, we selected CNCTC 10/84 to allow us to distinguish whether diabetes promotes increased GBS susceptibility by prolonging colonization in an otherwise short-term colonization model. Additionally, *de novo covRS* mutations are observed in a mouse diabetic wound model (60) and in human vaginal isolates (61), supporting the relevance for modeling host-GBS interactions in the diabetic reproductive tract. Compared to control mice, the HFHS-fed group displayed sustained CNCTC 10/84 burdens at 7 days post-infection (dpi) in the vaginal lumen, although no differences were seen at earlier time points (Fig. 1F and G). Furthermore, HFHS-fed mice displayed increased GBS burdens in vaginal, cervical, and uterine tissues at 7 dpi (Fig. 1G). To determine the impact of metabolic disease on glucose homeostasis, we quantified glucose in the reproductive tract prior to and following infection. Surprisingly, HFHS-fed mice had no difference in glucose concentration in the vaginal lumen at baseline or in vaginal or uterine tissues at 7 dpi (Fig. 1H and I). In contrast, cervical tissue glucose was significantly decreased in HFHS-fed mice at 7 dpi (Fig. 1I). At earlier stages of metabolic disease, there were no differences in colonization outcomes for mice that had only undergone 4 weeks or 8 weeks of diet regimen (Fig. S1A through D). There was also no difference in GBS outcomes for mice that underwent a full 12 weeks of diet regimen, but only 2 days of CNCTC 10/84 colonization (Fig. S1E and F). Infections were repeated with A909 after 12 weeks on diet, but no significant differences in GBS burden between groups were observed in the vaginal lumen or in any of the reproductive tract tissues (Fig. S1G and H). Together, these results indicate that the HFHS diet worsens the outcomes of GBS vaginal colonization in a manner dependent on metabolic disease duration, duration of colonization, and GBS strain.

### Elevated body mass gain and post-infection tissue glucose correlate with GBS burden

T2D manifests multiple measurable metabolic features, including impaired glucose tolerance, gain in body mass, and elevated glucose in tissues and fluids (62–64). To clarify the impact of metabolic disease versus the direct impact of diet on GBS outcomes, we sought to determine whether any metabolic features correlated with GBS burdens. Glucose tolerance prior to infection did not correlate with GBS burden at any swab time point nor in any reproductive tissues (Fig. 1J). However, we found that mice that gained more body mass had poor GBS outcomes, indicated by a positive correlation between percent increase in body mass and GBS burdens in 7 dpi swabs and tissues (Fig. 1K; Fig. S2A and B). To address the relationship between reproductive tract glucose and GBS colonization, we correlated glucose concentrations with CNCTC 10/84 burdens. Glucose levels in the vaginal lumen, taken pre-infection, did not correlate with GBS burden at any swab time point (Fig. S2C), indicating that vaginal glucose availability does not impact infection outcomes. At 7 dpi, glucose levels in vaginal tissue did not significantly correlate with vaginal tissue GBS burdens, but cervical and uterine glucose negatively correlated with GBS burdens at their respective sites (Fig. S2D). These data suggest that GBS glucose consumption is augmented in the cervix and uterus compared to the vagina or that glucose limitation benefits GBS spread to the cervix and uterus. The results also reveal no increase in glucose levels in the diabetic female reproductive tract, discordant with elevated levels seen systemically and at other mucosal sites (65, 66).

### Diabetic mice have minimal changes in the vaginal microbiome

**Fig 2:**
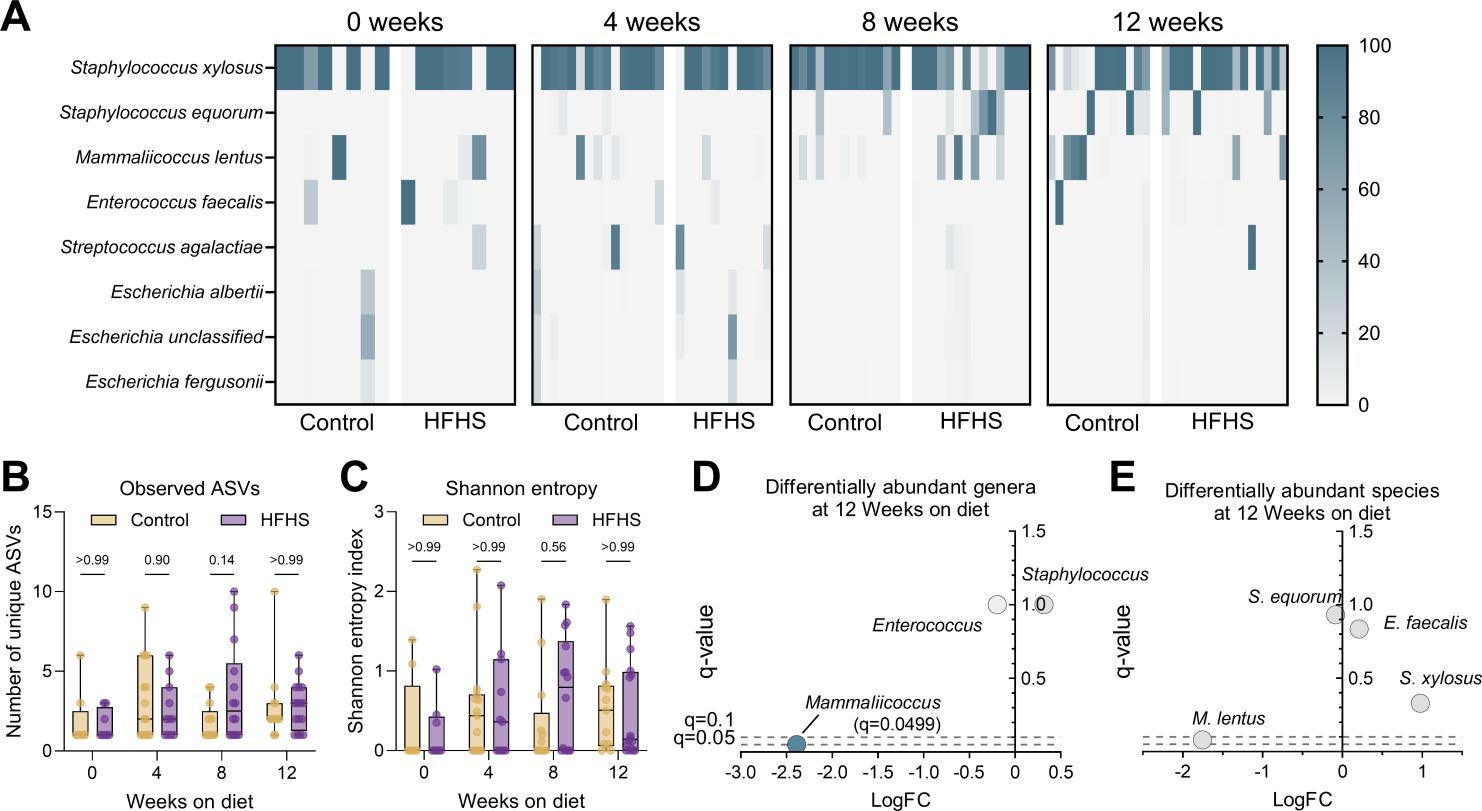
Twelve weeks of HFHS diet does not alter vaginal microbiome bacterial diversity but decreases the relative abundance of *Mammaliicoccus*. (**A**) Heatmap of relative abundances for bacterial genera/species in individual mice at 0, 4, 8, or 12 weeks on diet. (**B**) Total unique observed amplicon sequence variants (ASVs) in each group at 0, 4, 8, or 12 weeks on diet. (**C**) Shannon entropy indexes in each group at 0, 4, 8, or 12 weeks on diet. (**D**) Differentially abundant genera in the HFHS diet group compared to the control diet after 12 weeks. (**E**) Differentially abundant species in HFHS diet group compared to control diet after 12 weeks. *n* = 8–16 (**A–C**), *n* = 13–16 (**D and E**). Data represent two independent experiments. Heatmap columns represent individual mice (**A**). Box and whisker plots show all points, representing individual samples, lines indicate medians, and whiskers extend from minimum to maximum (**B and C**). Data were analyzed two-way ANOVA with Benjamini, Krieger, and Yekutieli correction and false discovery rate (FDR) set at 5% (**B and C**) or ANCOM-BC2 with diet as a fixed effect (**D and E**).

The vaginal microbiome is implicated in susceptibility to urogenital infection, but little is known about the vaginal microbiome in the context of T2D (40–43). To assess changes in the microbiome composition at successive stages of metabolic disease development, we profiled the vaginal microbiome of HFHS-fed and control mice at 0, 4, 8, and 12 weeks on the diet. Vaginal communities in both groups were primarily dominated by *Staphylococcus xylosus* at all time points, with less frequent dominant taxa including *Staphylococcus equorum, Enterococcus faecalis, Mammaliicoccus lentus, Streptococcus agalactiae* (GBS), and *Escherichia* spp. (Fig. 2A). The spontaneous emergence of GBS was not retained in any individual mouse across more than one time point, indicating that GBS presence in the vaginal microbiota of these mice was transient (Table S1). At all time points, no significant differences in alpha diversity metrics, including amplicon sequence variants (ASVs) or Shannon Entropy, were detected (Fig. 2B and C). At 4- and 8-week time points, no differentially abundant genera or species were detected (Table S1). At 12 weeks on the diet, differential abundance analysis via ANCOM-BC2 revealed a decrease in *Mammaliicoccus* genus in the HFHS-fed group (Fig. 2D). Two alternative methods, Mann-Whitney and mixed linear modeling, did not identify differentially abundant genera (Table S1). Additionally, no species or ASVs were significantly differentially abundant by any method (Fig. 2E; Table S1). Despite frequent sequencing- and cultivation-based detection of murine vaginal *Enterococcus* in prior studies using the same vendor and vivarium (36, 39, 67), in this study, detection of vaginal *Enterococcus* via sequencing was rare (Table S1), and no differences in vaginal swab or tissue *Enterococcus* detection rate or CFU were observed between diet groups (Fig. S3A through D). However, *Enterococcus* burden positively correlated with GBS burden at later time points (3 and 7 dpi) in vaginal swabs and in all three reproductive tissues at 7 dpi (Fig. S3E and F).

### Diabetic mice have aberrant vaginal cytokine responses to GBS

**Fig 3:**
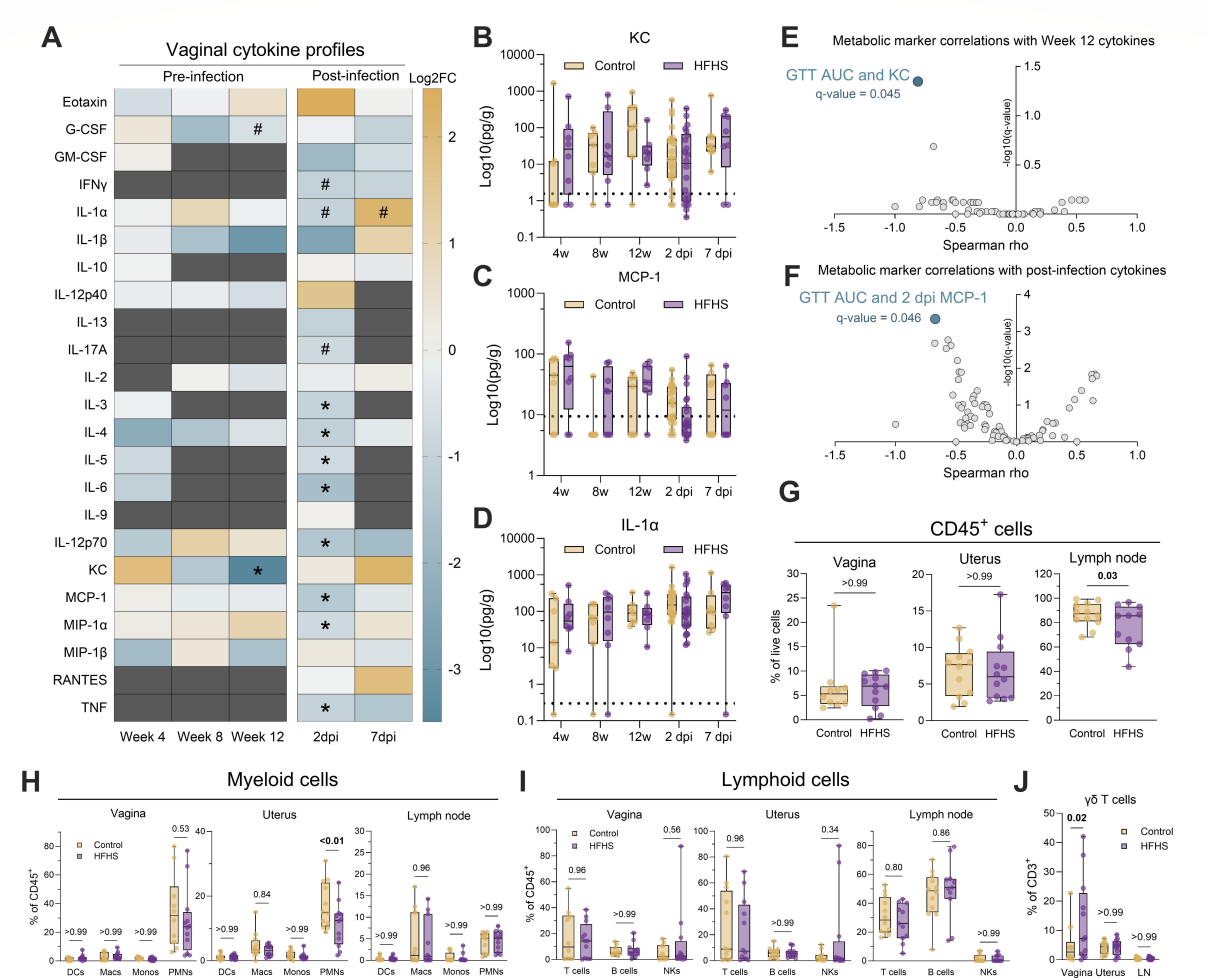
HFHS diet suppresses a baseline vaginal chemokine, broadly suppresses early vaginal cytokine response to GBS, and alters immune cell proportions. (**A**) Vaginal lavage fluid cytokine profiles pre-and post-infection with CNCTC 10/84, displayed as log2FC of HFHS group median over control group median. Dark gray indicates timepoints where at least one group had less than two samples meeting the lower detection limit of that cytokine. Vaginal lavage concentrations of KC (**B**), MCP-1 (**C**), and IL-1α (**D**) normalized to total protein. Spearman correlation results between metabolic markers and baseline (**E**) or post-infection (**F**) vaginal cytokine concentrations. (**G**) Proportion of CD45^+^ cells out of total live cells at 2 days post-infection (dpi) in the vagina, uterus, and ileal lymph node (LN). Myeloid (**H**) and lymphoid (**I**) cell proportions out of CD45^+^ cells at 2 dpi. Cell populations were defined as follows: dendritic cells (DCs, CD11b^var^, CD11c^var^, MHC-II^+^, and CD24^+^), macrophages (Macs, CD11b^var^, CD11c^var^, MHC-II^+^, and CD64^+^), monocytes (Monos, CD11b^var^, CD11c^var^, MHC-II^-^, and Ly6C^+^), neutrophils (PMNs, CD11b^var^, and Ly6G^+^), T cells (CD3^+^), B cells (CD19^+^), and natural killer cells (NKs, NK1.1^+^, and CD11b^var^). (**J**) Proportions of γδ T cells (CD3^+^, TCRγδ^+^) out of CD3^+^ cells at 2 dpi. Gating scheme defining markers of immune cell subsets is provided in Fig. S5. *n* = 7–16 (**A–D**) or *n* = 10–12 (**G–J**). Cytokine data represent 1 (pre-infection and 7 dpi time points) or 3 (2 dpi time point) independent experiments. Immune cell data represent four independent experiments. Box and whisker plots show all points, representing individual samples, lines indicate medians, and whiskers extend from minimum to maximum, with dotted lines indicating the lower limit of detection (**B–D**). Data were analyzed by two-tailed Mann−Whitney *U* test (**A–D, G**), Spearman correlation with Benjamini-Hochberg FDR correction (**E and F**), or two-way ANOVA with Šidák correction for multiple comparisons (**H–J**). For panel A, ^#^*P* < 0.1, *P < 0.05.

Another potential mechanism by which type 2 diabetes may confer increased susceptibility to GBS is through alterations to reproductive tract immune responses. To test this possibility, we quantified 23 cytokines in vaginal lavages collected by washing the vaginal tract with PBS at 4, 8, and 12 weeks on diet as well as 2 dpi and 7 dpi, time points with established innate immune induction in this model (44, 45). Cytokines that were detected in at least two samples per group were retained for analysis for each timepoint (Fig. S4; Table S2). While no significant differences between diet groups were seen at 4 or 8 weeks on diet, by week 12, HFHS-fed mice displayed lower levels of chemokine KC compared to control mice (Fig. 3A and B; Fig. S4). At 2 dpi, eight cytokines were significantly lower in the HFHS-fed group compared to the control group, including MCP-1 (Fig. 3A and C). Several additional cytokines exhibited a potential reduction, but comparisons were not statistically significant (IL-1α, *P* = 0.06; IL-17A, *P* = 0.06). No significant cytokine differences were observed at 7 dpi, although GBS burdens were higher in HFHS-fed mice at that time point, but we did note differential IL-1α fluctuation between HFHS-fed and control mice over the course of infection (Fig. 3D). We then correlated cytokine levels with metabolic markers of glucose intolerance, body mass, and weight gain. Glucose intolerance negatively correlated with pre-infection KC levels at 12 weeks on diet and with post-infection MCP-1 levels at 2 dpi (Fig. 3E and F). Additionally, we observed a positive correlation between 4-week MCP-1 and 12-week GTT AUC and an inverse correlation between 4-week IL-4 and 12-week GTT AUC, but these findings did not achieve statistical significance (Table S2). Taken together, these results indicate that the HFHS diet impairs local vaginal immune signaling in response to GBS challenge and suggest broad impacts on pathways relating to both innate and adaptive immunity.

### Diabetic mice have reduced uterine neutrophil proportions in mock and GBS-exposed groups and enriched vaginal γδ T cells during GBS challenge

The aberrant vaginal cytokines we observed at baseline and in response to GBS suggest that diabetes may impair the recruitment, activation, and/or function of immune cells in the reproductive tract. To assess immune cell populations during infection, we harvested the vagina and uterus at 2 dpi and performed flow cytometry. We also harvested ileal lymph nodes, which drain the pelvic region, including the vagina and uterus (68). A panel of 17 markers was used to detect myeloid lineages, lymphoid lineages, and nine T cell subsets (Table S2; Fig. S5A). Aggregate lymphocyte (CD45^+^) proportions were comparable between diet groups in the vagina and uterus; however, HFHS-fed mouse lymph nodes had a modest, but significant, reduction (1.9% decrease) in the CD45^+^ proportion of live cells compared to controls (Fig. 3G). This reduction could not be attributed to a specific cell type (Fig. 3H and I). We observed decreased uterine neutrophil proportions among CD45^+^ cells (polymorphonuclear cells, PMNs) in HFHS-fed mice compared with controls (Fig. 3H). Across tissues, other myeloid and lymphoid lineages did not significantly differ between groups at 2 dpi (Fig. 3H and I). Most T cell subsets showed no significant difference in any tissue, except for vaginal γδ T cells, which showed elevated proportions among CD3^+^ cells in HFHS-fed mice (Fig. 3J; Fig. S5B through D). Finally, in mock-infected mice, no significant differences were observed for any cell type, with the exception of reduced proportions of uterine neutrophils in the HFHS-fed mice (Fig. S5E through J). Collectively, these results suggest that diabetes results in abnormalities of reproductive tract immune populations at baseline and in response to GBS challenge.

### Exogenous IL-1α treatment ablates diabetic susceptibility to GBS

**Fig 4:**
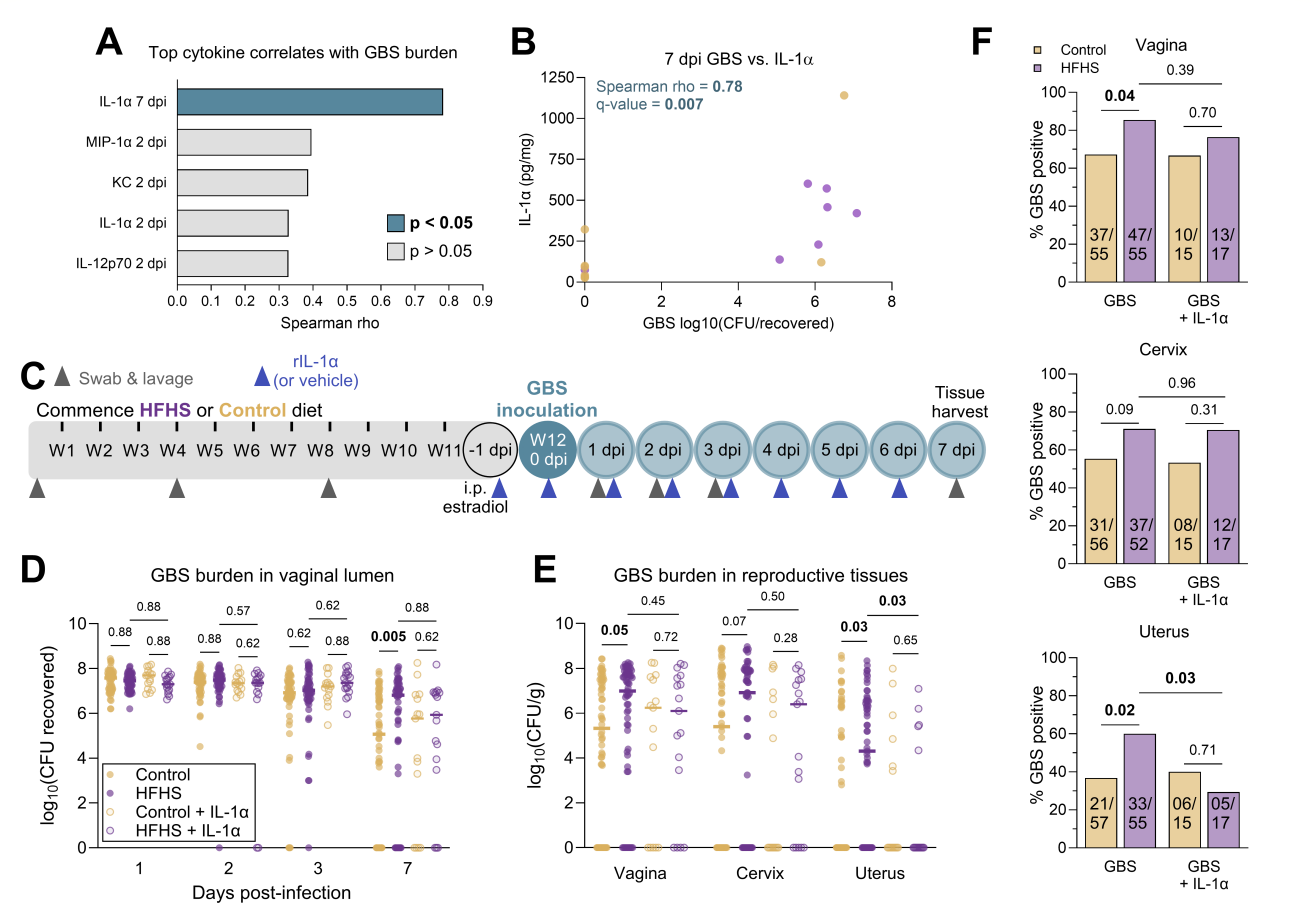
IL-1α supplementation resolves elevated endpoint GBS burdens and uterine ascension in HFHS-fed mice. (**A**) Spearman correlations between vaginal cytokine concentrations and vaginal GBS CNCTC 10/84 burdens at paired time points. Cytokines with <3 detectable values per diet group were excluded, and the cytokines with the five strongest rho values are displayed. (**B**) In total, seven dpi vaginal swab GBS burdens plotted with 7 dpi vaginal IL-1α. (**C**) Experimental timeline of type 2 diabetes development on HFHS diet and subsequent rIL-1α supplementation (or carrier) paired with GBS vaginal colonization. (**D**) GBS burdens from post-infection vaginal swabs. (**E**) GBS burdens from post-infection reproductive tissues. (**F**) Proportions of mice in each group with GBS detected in the uterine tissue. *n* = 7–8 (**A and B**), *n* = 15–57 (**D–F**). Data in panels D and E represent data from Fig. 1F and G, shown for comparison with rIL-1α treatment groups. Data represent 1 (**A and B**), 2 (**D–F**, groups receiving rIL-1α), or 8 (**D–F**, groups not receiving rIL-1α, two of which were performed in tandem with rIL-1α treatment) independent experiments. Data were analyzed by Spearman correlation with Benjamini-Hochberg FDR correction (**A and B**), two-way ANOVA with Benjamini, Krieger, and Yekutieli correction and false discovery rate (FDR) set at 5% (**D and E**), or two-sided Fisher’s exact test (**F**).

We next sought to confirm whether specific vaginal cytokine deficiencies contribute to the increased susceptibility to GBS seen in diabetic mice. To clarify which cytokines were linked to infection outcomes, we performed Spearman correlations between GBS burden and cytokine concentrations at paired time points. IL-1α at 7 dpi was the only significant cytokine that correlated with GBS CFU (Fig. 4A and B; Table S2). Because of this connection between IL-1α and GBS burden, and the general pattern of reduced cytokines in HFHS-fed mice, we selected this broadly acting cytokine for an intervention study. We supplemented mice with topical vaginal recombinant IL-1α or vehicle (PBS) beginning 1 day prior to GBS infection and daily throughout the infection time course (Fig. 4C). Vehicle treatment did not alter GBS vaginal lumen or tissue burdens (Fig. S5K and L); thus, nontreated groups were included in analyses. Treatment with IL-1α abrogated differences in GBS burdens between diabetic and control groups in the vaginal lumen and vaginal and uterine tissues at 7 dpi (Fig. 4D and E), and absolved differences in GBS detection rates in vaginal and uterine tissues (Fig. 4F). Furthermore, within HFHS-fed mice groups, IL-1α treatment decreased uterine GBS burdens and detection rates compared to HFHS-fed mice who did not receive IL-1α (Fig. 4F). Taken together, these results indicate that increased GBS colonization and dissemination in diabetic mice can been absolved by supplementing with IL-1α at the site of colonization.

## DISCUSSION

Although type 2 diabetes is a leading risk factor for GBS invasive disease in non-pregnant adults (5), the biological drivers of this increased susceptibility are not fully known. In this study, we found that a diet-induced murine model of T2D exhibits increased GBS vaginal persistence and ascension to the upper reproductive tract. The vaginal microbiome was minimally altered in diabetic mice, suggesting that changes in species-level microbiome composition have a negligible impact on GBS outcomes in this model. Conversely, we found that HFHS-fed mice failed to mount a vaginal pro-inflammatory response to GBS and that certain cytokine responses were delayed. We further showed that pro-inflammatory cytokine supplementation early during colonization resolves the heightened susceptibility to GBS in HFHS-fed mice. Together, these findings reveal impaired mucosal immune responses and enhanced pathogen colonization as potential precursors of GBS-invasive disease in diabetic individuals.

We selected a diet-induced model as a relevant simulation of T2D, considering the majority of T2D cases are attributable to diet (69), and there is an increasing global prevalence of highly processed diets high in fats and sugars (70). Clinically, obesity is a risk factor for GBS vaginal colonization (71, 72). Although we observed significantly increased body mass at 4 and 8 week time points, we did not detect any differences in microbiota composition, GBS susceptibility, or cytokine profiles, suggesting limited phenotypes associated with obesity prior to diabetes onset in this model. Using a 7-week course refined diet model, Megli et al. recently described increased GBS persistence in mice fed any refined diet—either high fat, low soluble fiber or low fat, low soluble fiber—compared to regular chow (21). Similar to Megli et al., we found no correlations between glucose intolerance and GBS burdens; however, divergently, we found that body weight gain significantly correlated with GBS vaginal levels at day 7. There are several experimental differences that may explain this discordance. First, the length of dietary exposure (7 week vs. 12 week) may accentuate phenotypes in our study. Additionally, Megli et al. supplied experimental diets over puberty, whereas our study exclusively treated mice post-puberty, which may have altered the extent of metabolic disease. Finally, dietary formulations, although similar in terms of fiber (5%—6%), differed in terms of high-fat (60% vs. 45% energy from fat) and sucrose content (9% vs. 21%) between the study and this study, respectively. Collectively, these findings suggest that both dietary components and metabolic metrics impact GBS susceptibility, aligned with clinical observations of obesity and sugary drink intake as risk factors for GBS vaginal colonization (73–75).

Elevated hemoglobin A1c (HbA1c), an indicator of sustained hyperglycemia, is associated with increased risk for vaginitis and genital infections, including HPV and Candidiasis (76–78), as well as colonization by pathogens, including GBS and other pathobionts (20, 79, 80). Although diabetic patients display elevated glucose in mucosal secretions such as saliva and tears (65, 81, 82), few citations exist wherein diabetic vaginal glucose is empirically measured (24, 25). One human study quantified vaginal glucose in primarily non-diabetic patients before and during glucose tolerance testing and discovered a surprising decrease in vaginal glucose after the oral glucose bolus, independent of HbA1c, plasma glucose, or BMI (24). These findings suggest that, during a hyperglycemic state, excess glucose in the vaginal mucosa is effectively metabolized or stored, reducing its detection in the lumen. Further studies are needed to determine the impact of hyperglycemia on the fate of glucose in the reproductive tract. Excess glucose may accumulate inside differentiated keratinocytes (83), a phenomenon which could select for pathogens with capacity for cytotoxicity or intracellular invasion. Alternatively, it may be metabolized by microbiota directly or after conversion to glycogen (84) or non-enzymatically incorporated into advanced glycation end products, with myriad downstream impacts on tissue physiology and architecture (85, 86).

In our model, vaginal lavage and tissue glucose levels were similar in diabetic and control mice. Moreover, cervical tissue glucose levels were decreased in HFHS-fed mice. This may be attributable to GBS utilization of glucose, as GBS burdens negatively correlated with glucose concentrations in the cervix. However, the same negative correlation between GBS and glucose was observed in uterine tissue, despite glucose levels being similar in the uterine tissue of diabetic and control mice. Compared to vaginal and cervical glucose levels, uterine glucose levels were lower overall. These disparate findings in the lower versus upper reproductive tract suggest that GBS may alter its metabolism as it adapts to tissue niches. In line with this, multiple GBS carbon utilization genes are differentially expressed between vaginal and uterine tissues in gestational diabetic mice compared to controls, including a putative GBS glycosyltransferase *yfhO* critical for GBS uterine ascension (39). In contrast to our findings, increased vaginal glucose was reported in a type 1 diabetic mouse model (26), suggesting alternative physiological impacts on tissue glucose homeostasis across diabetic manifestations. It is worth noting that a wide range of glucose concentrations was observed in control mice in the vagina and cervix; however, these ranges were similar to previously published ranges in pregnant mice on the same diets (39). Although not evaluated in the context of our model, biological factors such as epithelial turnover rates, estrogen variability due to drifts in estrous stages, or the metabolic functional capacities of the vaginal microbiome may contribute to this variability (84, 87–89).

The human vaginal microbiome has been profiled in gestational diabetes mellitus (90–93), and more recently in T2D (40–43). Although there is heterogeneity in specific taxonomic findings, collectively these studies suggest diabetes-related reduction in health-associated *Lactobacillus* spp. and enrichments in infection-associated bacterial taxa. Some of these taxa, such as *Enterococcus* and *Escherichia*, co-occur with GBS *in vivo* (39, 94–97) and have the capacity to behave synergistically with GBS *in vitro* (98). Their enrichment may partially explain the increased susceptibility to GBS colonization in diabetic patients (9, 20). A key limitation of murine models is the inability to replicate the vaginal community composition of humans, although overall low alpha-diversity and dominance by one or two taxa are shared between species (36, 37, 67). Megli et al. used a conventional mouse model to investigate microbiome dynamics before and during long-term GBS vaginal colonization, wherein they found that mice on a high-fat diet had increased vaginal *Streptococcus* and *Enterococcus* and decreased *Staphylococcus* at baseline and at multiple post-infection time points (21). HFHS-fed mice in our current study had decreased relative abundance of *Mammaliicoccus*, a close relative to *Staphylococcus* in the family *Staphylococcaceae*, suggesting shared constraints on a similar microbial niche. However, *Streptococcus* and *Enterococcus* were not detected in many of our mice. Demirel et al. profiled vaginal microbiomes of hyperglycemic rats and found no significant changes (99). These dissimilar findings reflect that inherent differences in species, vendors, and/or animal facilities play a role in bacterial compositions and thus influence microbiome findings downstream. Despite modest findings related to the vaginal microbiome composition, we cannot rule out diabetes-related changes to microbial metabolic capacity and other functions. Our findings would be complemented by microbiota depletion studies to more fully interrogate the potential contribution of the vaginal microbiota to GBS colonization outcomes in diabetes.

T2D is often considered a chronic inflammatory condition, with sustained elevation of immune markers such as C-reactive protein and IL-6 (48, 100). Other studies have shown the predictive power of pro-inflammatory and immunoregulatory cytokine levels for a future T2D diagnosis (48, 101–104). While systemic baseline inflammation is well-documented, T2D impacts on mucosal immune responses, particularly in the context of infection, are more sparsely described. When we profiled vaginal cytokines in our HFHS mouse model, we discovered a broad suppression in many pro-inflammatory cytokines compared to control mice. These effects appeared as early as 4 weeks on diet and were most prominent during the GBS challenge. When we profiled immune cells in the reproductive tract and ileal lymph nodes during GBS challenge, we found that total immune cell proportions were slightly lower in HFHS lymph nodes versus control. Additionally, we found that HFHS-fed mice displayed reduced proportions of uterine neutrophils, previously reported to contribute to effective reproductive tract clearance of GBS in both non-pregnant murine models (47) and pregnant fetal tissues (39). This difference was noted both in mock and GBS-challenged diabetic groups; however, our low sample size in the mock group (*n* = 3) and the lack of differences in uterine GBS burdens at the 2 dpi time point warrant that these results be interpreted with caution. We also found that vaginal γδ T cell proportions were expanded in HFHS-fed mice compared to controls. This was somewhat surprising, given that γδ T cells aid in clearing vaginal GBS (47), and we observed persistence of vaginal GBS in HFHS-fed mice. This finding may potentially represent an expansion of the vaginal γδ T cell compartment that is compensatory to exhaustion. In humans, type 2 diabetes is associated with reduced peripheral γδ T cells that display elevated exhaustion markers compared to non-diabetic patients, and obesity positively correlates with exhaustion marker co-expression (105). The HFHS-fed mouse cytokine profiles also support a potential γδ T cell exhaustion phenotype. γδ T cells rapidly produce IL-17A in response to infection, and they are a primary source of IL-17A in the reproductive tract (47, 106, 107). Despite this, we did not observe elevated IL-17A at any time point. Rather, we observed a non-significant decrease in vaginal IL-17A at 2 dpi, a time point at which γδ T cells were enriched. This evidence points to a population of γδ T cells that have potentially become functionally deficient; however, this must be followed up with exhaustion marker staining and intracellular IL-17 staining, γδ T cell depletion, and *ex vivo* functional assays.

Our observed cytokine phenotypes indicate that immune impairment develops over the course of metabolic disease progression in the T2D vaginal tract. KC and MCP-1 are implicated in the development of obesity and insulin resistance (108, 109), and we observed a positive, but non-significant, correlation between 4-week MCP-1 and subsequent glucose intolerance. At later time points, we observed inverse correlations between vaginal levels of these chemokines and glucose intolerance. It is also possible that diabetes alters immune response kinetics. GBS stimulates robust IL-1α induction at the uroepithelium (110, 111), gravid reproductive tract (39), and choriodecidua (112), and here, we found that GBS burdens were highly correlated with vaginal IL-1α at 7 dpi, primarily driven by diabetic mice. When we supplemented with exogenous rIL-1α, heightened susceptibility to GBS was ablated in HFHS-fed mice and uterine ascension was limited, suggesting that immune stimulation is sufficient to improve vaginal GBS colonization outcomes in diabetic mice. IL-1α is known to contribute to reproductive tract neutrophil recruitment (113), which may explain the reduced uterine GBS burdens in IL-1α-supplemented diabetic mice. IL-1α and other IL-1 family effector cytokines can also stimulate γδ T cell function, namely IL-17A production, in response to a pathogen (114–116). Exogenous IL-1α supplementation may have pushed exhausted γδ T cells toward effector-like function via direct interaction or through its ability to stimulate IL-1 family signaling in a positive feedback loop (116, 117), although this remains to be experimentally confirmed. IL-1α may have also modulated glucose metabolism, with downstream impacts on immune cell or epithelial cell functions. Glucose metabolism and IL-1α production have been linked in other tissues; IL-1α increases glucose uptake in muscle (118), and elevated glucose stimulates IL-1α production in renal epithelial cells (119). In invasive infection with closely related group A *Streptococcus*, IL-1α coordinated liver metabolic adaptation and tolerance to infection (120); however, the impact of IL-1α, whether endogenous or exogenous, on reproductive tract tissue glucose homeostasis and downstream effects on immunity remains uncharacterized. Longitudinal profiling of systemic and local metabolism and immunity and immune cell behavioral assays (e.g., chemotaxis/killing) are needed to confirm these observations.

Our results may be sex-specific or specific to certain epithelial sites. Of the few studies that investigated sex differences in systemic diabetic inflammation, in adolescent humans with T1D and a rat model of T2D, only males exhibit increases in inflammatory cytokines, whereas females exhibit decreases in these same inflammatory cytokines (121, 122). Furthermore, a study on female mini-pigs showed that long-term high-fat diet lowered serum IL-1β, IL-10, and IL-4, suggesting a general decrease in both pro- and anti-inflammatory cytokine-mediated immune signaling (123). Regarding site specificity, diabetic mouse models of GBS infection generate distinctive inflammation responses depending on the infected tissue. In a T2D urinary tract infection (UTI) model, both male and female db/db mice had suppressed pro-inflammatory cytokines and reduced immune cell infiltration in the bladder (52). In contrast, models of diabetic lung, wound, arthritis, T1D UTI, and systemic infection show that diabetic mice have increased pro-inflammatory cytokines and immune cell infiltration or upregulated immune cell infiltration pathways (51, 60, 124, 125). Finally, it is possible that multiple host metabolic factors shape the mucosal immune landscape in diabetes, leading to this collection of diverging results among distinct diabetic disease types and host states. Indeed, gestational diabetic mice of the same genetic background and fed the same diet as this current study, when compared to non-diabetic pregnant controls, displayed several increased inflammatory vaginal cytokines, elevated vaginal B cell and uterine NK cell recruitment, and decreased uterine T_Reg_ proportions upon GBS challenge (39).

It is necessary to note the limitations of our study. First, diet-induced models of metabolic disease cannot untie the impacts of dietary components from the impacts of metabolic disease. With its long-term 12-week diet time course, our study design minimizes the impact of early host and microbiota adaptation to dietary components. Our study would be complemented by replication studies using genetically predisposed (e.g., db/db) or chemically induced (e.g., STZ) models, such as those used to study GBS urinary tract infection (51, 52). Second, our experiments were conducted primarily with GBS strain CNCTC 10/84 (serotype V), which demonstrated increased colonization in diabetic mice compared to controls, whereas another strain, A909 (serotype Ia), did not exhibit differential colonization. These disparate findings are observed despite the relevance of both serotype V and serotype Ia to diabetic infection (56–58). Thus, our findings may only be applicable to certain GBS genetic backgrounds. CNCTC 10/84 is generally considered a hyper-virulent strain due to CovRS deficiency and hyper-hemolytic activity and, interestingly, is rapidly cleared from the murine vaginal tract (55). Notably, *covR* deletion in the A909 background also reduces vaginal GBS persistence and elevates local immune responses to colonization (44). Our observation of prolonged CNCTC 10/84 persistence in the diabetic vagina, when the strain is typically readily cleared in conventional mice prior to 7 dpi, supports that weakened diabetic immunity may promote vulnerability to pathogens that may otherwise pose a limited threat. Third, our microbiome profiling experiments are constrained due to the limited suitability of the murine vaginal microbiome as a model of the human vaginal microbiome. Although the majority of human vaginal microbiomes are dominated by a *Lactobacillus* species, conventional mice are very rarely dominated by any *Lactobacillus* or closely related genera (36, 37). The potential to determine vaginal microbiome phenotypes of type 2 diabetes will likely be greatest in human studies and in humanized models of the vaginal microbiome (36, 126). Finally, we have identified a potential sex difference or tissue specificity in diabetic immune phenotypes, but our all-female study design and focus on the female reproductive tract inherently precludes traditional sex-based comparisons. Future studies should also investigate mechanisms of sex differences in the diabetic immune response, including sex hormones and genetic components.

In summary, we showed that dysregulated immune responses to group B *Streptococcus* at the vaginal mucosa contribute to increased bacterial burdens during colonization. Our findings provide an explanation for increased GBS vaginal colonization in type 2 diabetic patients and highlight that deficient immunity may be an important but underexplored phenotype in females and/or at mucosal sites. Future investigation should focus on the role of γδ T cells in diabetic vaginal GBS colonization and on pharmaceutical immune modulation approaches to control GBS colonization in the vulnerable diabetic host. Furthermore, because type 2 diabetic patients are susceptible to an array of urogenital pathogens, the impact of diabetes-associated immune suppression should be investigated with regard to other taxa, such as *Klebsiella, Escherichia coli,* and *Candida* spp. In this population, immunostimulatory therapies may be a viable alternative to antibiotic-based treatments in the age of increasing recurrent infection and antibiotic resistance.

## MATERIALS AND METHODS

### Animals

Female wild-type C57BL/6J mice from Jackson Laboratories (strain code 000664) were used for all experiments. Mice were housed at a maximum of four animals per cage and given food and water *ad libitum*. A 12-h light cycle was used. Mice were purchased at 6 weeks old and randomly assigned to a diet group. The two groups were fed a high-fat high-sucrose (HFHS) diet (D12451, Research Diets Inc., 45% kcal fat, 17% kcal sucrose) or a control low-fat no sucrose diet (control) (D12450K, Research Diets Inc., 10% kcal fat, <1% kcal sucrose) for up to 12 weeks prior to GBS colonization, until the experimental endpoint, up to a total of 13 weeks. A subset of mice was originally used for timed pregnancy experiments and were cohabitated with males for 3 days after 1 week on diet. Only mice that did not become pregnant were used for the present study.

### Assessment of metabolic markers

To determine body mass, mice were weighed on a scale at baseline prior to diet and then at 4 weeks, 8 weeks, and 12 weeks after starting diet. To determine fasting blood glucose, food and water were withheld for 4 h, then blood glucose was measured using a glucometer (ReliOn Prime Blood Glucose Monitoring System) via tail venipuncture. To determine glucose tolerance, food and water were withheld for 4 h, then baseline fasting blood glucose was measured at the 0-min time point. Mice were then given an intraperitoneal injection of 1 mg glucose in PBS per 1 g body mass in a 100 µL volume. Blood glucose was then measured at 15, 30, and 90 min post-glucose bolus.

### GBS vaginal colonization

Mice were colonized as described previously at 4, 8, or 12 weeks after beginning the diet (55). One day prior to GBS infection, mice were injected intraperitoneally with 0.5 mg 17 β-estradiol (Sigma-Aldrich) suspended in 100 µL sesame oil. *Streptococcus agalactiae*, or group B *Streptococcus* (GBS), was grown in Todd-Hewitt broth (THB) (Hardy Diagnostics, Santa Maria, CA) at 37°C in aerobic, static conditions. Wild-type clinical GBS isolates CNCTC 10/84 (ATCC 49447, serotype V) or A909 (ATCC BAA-1138, serotype Ia) were grown overnight in 3 mL THB, then sub-cultured by adding 0.3 mL overnight culture to 2.7 mL fresh THB, and grown to log phase at an OD600 of 0.4–0.6. GBS was pelleted then resuspended in PBS. Each mouse was inoculated vaginally with 10^7^ CFU of GBS suspended in 10 µL using gel-loading pipette tips as described previously (55).

### GBS quantification in the vaginal lumen

Vaginal swabs were collected as described previously (55). Briefly, sterile nasopharyngeal swabs (Puritan) were placed in 100 µL sterile PBS to wet the swab tip, then inserted into the vaginal opening. Swabs were rotated four times clockwise and four times counterclockwise and then removed and placed back in sterile tubes of PBS. Blank samples were generated during each sample collection by exposing a sterile swab to the laminar air flow hood environment for several seconds and then re-submerging the swab in the tube of PBS and processing with identical methods as other swab samples. Swabs were secured inside tubes, and tubes were vortexed for 30 s to dislodge bacteria from the swab tip. Samples (10 µL) were used for serial dilution and plating on selective chromogenic agar media (CHROMAgar StrepB, DRG International, Inc.). Purple/mauve colonies were counted to quantify GBS CFU, and dark blue colonies were counted to quantify *Enterococcus* CFU.

### GBS quantification in reproductive tissues

At the experimental endpoint, mice were sacrificed, and the vagina, cervix, and uterus were harvested from each mouse. Whole tissues were placed in pre-weighed 2 mL screwcap microcentrifuge tubes containing 500 µL PBS and 1.0 zirconia-silicate beads. Tubes were weighed again after the addition of tissues and stored in ice until placed in a Roche Magnalyser bead beater. Tissues were homogenized at 6,000 rpm for 60 s; 10 µL of each homogenized tissue sample was used for serial dilution and plating on CHROMAgar StrepB. Purple colonies were counted to quantify GBS CFU, and dark blue colonies were counted to quantify *Enterococcus* CFU. Cardiac blood was harvested from each mouse and plated undiluted on CHROMAgar StrepB to confirm the absence of GBS bacteremia.

### Vaginal microbiota 16S rRNA sequencing

Vaginal lumen swabs were collected as described above, and samples were stored at −20°C until microbial DNA was extracted using Quick-DNA Fungal/Bacterial Microprep Kit (Zymo Research). DNA was eluted in 20 μL molecular biology grade water. At SeqCoast Genomics (Portsmouth, NH, USA), a sequencing library was generated by amplifying the v3/v4 region of the 16S rRNA gene via PCR using the primer pair 341F and 806R using the Zymo Quick-16S Plus NGS Library Prep Kit. The DNA library was sequenced on an Illumina NextSeq2000 platform using a 600-cycle flow cell kit to produce 2 × 300 bp paired reads; 30%–40% PhiX control (unindexed) was spiked into the library pool to support optimal base calling of low diversity libraries on patterned flow cells. Read demultiplexing, read trimming, and run analytics were performed using DRAGEN v4.2.7 software on the NextSeq2000. Reads were combined into a single FASTA file, and the rest of the sequencing analysis was performed in QIIME2 v2024.2 unless otherwise noted. Sequences were joined and trimmed to 300 bp and then denoised using the DADA2 plugin. OTUs were assigned taxonomy by mapping reads to the Greengenes2 database v 2022.10 with a similarity cutoff of 99%. Decontamination was performed by filtering out ASVs that appear in blanks or in <5% of samples and removing known contaminants from the DNA extraction process (Table S1). Decontaminated sequences were returned to QIIME2 for community diversity analyses.

### Quantification of cytokine levels in vaginal lumen

Pre-infection vaginal lavages were collected at 4, 8, or 12 weeks on diet immediately before GBS infection (0 dpi). Post-infection vaginal lavages were collected at 2 dpi or 7 dpi. Lavages were performed by pipetting 10 µL sterile PBS into the vagina of each mouse, pipetting four times, then moving the sample to a microcentrifuge tube, and diluting 5-fold in sterile PBS. Lavage samples were subjected to a 23-plex ELISA assay (Bio-Rad, Cat. No. M60009RDP) to quantify IL-1α, IL-1β, IL-2, IL-3, IL-4, IL-5, IL-6, IL-9, IL-10, IL-12 (p40), IL-12 (p70), IL-13, IL-17A, eotaxin, G-CSF, GM-CSF, IFN-γ, KC, MCP-1, MIP-1α, MIP-1β, RANTES, and TNF-α. Samples were thawed on ice, then centrifuged at 10,000 × *g* for 10 min. Supernatant was diluted 1:10 in Bio-Rad sample diluent and then processed according to manufacturer protocol. Fluorescence was detected on a Luminex MAGPIX instrument, and data were generated with Luminex xPONENT software for Magpix, version 4.2 build 1324. Data were analyzed with Milliplex Analyst, version 5.1.0.0 standard, build 10/27/2012. Log curves were fit to the mean fluorescent intensity data to calculate cytokine concentrations, with bovine serum albumin (BSA) standards as a reference. Cytokine data were normalized to total protein levels measured from each sample using a BCA assay (Pierce).

### Exogenous IL-1α intervention experiments

Mice were vaginally inoculated with 80 pg of recombinant mouse IL-1α, carrier-free (BioLegend, Cat. No. 575002) resuspended in 10 µL sterile PBS. Mice were treated every day beginning 1 day prior to infection, with the last treatment the day prior (6 dpi) to the endpoint. Mock intervention treatment was vaginal inoculation with 10 µL sterile PBS. All mice were then subjected to GBS vaginal colonization and GBS burden monitoring as described above.

### Immune cell profiling

Vaginal tissues were transected vertically, and one-third of the total tissue was used for GBS burden quantification, with the remaining two-thirds used for immune cell profiling. Uterine horns were transected horizontally, and one-half from each horn was pooled to generate one sample for a total of two samples. One uterine tissue sample was used for burden quantification and the other for immune cell profiling. Entire ileal lymph nodes (1–2 per mouse) were harvested and used for immune cell profiling. Sample processing for burden quantification was performed as described above, and samples for immune cell profiling were placed in 450 µL (vagina and lymph nodes) or 900 µL (uterus) of cold RPMI and kept on ice for the remainder of processing. Lymph nodes were cut into fourths with scissors, and vaginal and uterine tissues were diced with scissors until an estimated 90% of tissue particles were small enough to pipet through a 1,000 µL tip. Tissues were then enzymatically digested with collagenase (1 mg/mL) and DNAse (0.05 mg/mL) at 37°C, 250 rpm shaking for 30 min. Tissue pellets were briefly allowed to settle at the bottom of the tube; then, supernatant was filtered through 40 µm mesh cell strainers (uterus and lymph nodes) or Flowmi 40 µm pipet tip filters (vagina) into a tube with 800 µL of cold RPMI + 10% FBS. The remaining tissue particles from the pellet were supplemented with 350 µL of fresh RPMI and new doses of collagenase and DNAse, given in the same concentration as before. These samples then underwent a second enzymatic digestion incubation period with the same conditions as before. The supernatant was filtered and combined with the previously filtered supernatant. Cells were washed by centrifuging at 500 × *g* for 10 min, decanting RPMI, resuspending in 1 mL PBS, centrifuging, and decanting PBS. The cell suspension was concentrated by resuspending in 100 µL PBS. For viability staining, cells were centrifuged again and resuspended in 50 µL of diluted Zombie Aqua (1:1,000) and then incubated for 20 min at RT in the dark. Viability dye was washed off by adding 150 µL PBS and then centrifuging and decanting the supernatant. Blocking was performed by resuspending in 50 µL Fc block (CD16/CD32, clone 2.4G2, BD Biosciences, Cat. No. 553141, 0.5 mg/mL) diluted 1:200 in FACS buffer (PBS, 1 mM EDTA, 1% FBS, 0.1% sodium azide), then incubated 20 min at 4°C in the dark. Centrifugation was repeated, supernatant was decanted, then cells were resuspended in 100 µL of antibody cocktail overnight at 4°C in the dark. Antibody cocktail was suspended in brilliant stain buffer (Cat. No. 566385, BD Biosciences) and included 18 antibodies, which are listed in Table S2. After staining, samples were washed with 100 µL PBS, centrifuged, and resuspended in 150–300 µL FACS buffer, depending on the visual size of the pellet. Data were acquired on a BD FACSymphony A5 cytometer, and compensation and gating analyses were performed with FlowJo v10.10.1 software. The gating scheme is shown in Fig. S5A. Cell populations were defined as follows: lymphocytes (CD45^+^), T cells (CD3^+^), B cells (CD19^+^), natural killer cells (NKs, NK1.1^+^, and CD11b^var^), dendritic cells (DCs, CD11b^var^, CD11c^var^, MHC-II^+^, and CD24^+^), macrophages (Macs, CD11b^var^, CD11c^var^, MHC-II^+^, and CD64^+^), monocytes (Monos, CD11b^var^, CD11c^var^, MHC-II^–^, and Ly6C^+^), neutrophils (PMNs, CD11b^var^, and Ly6G^+^), CD4 T cells (CD3^+^ and CD4^+^), CD8 T cells (CD3^+^ and CD8^+^), CD4^+^ naive T cells (CD4^+^ and CD62L^+^), resident memory T cells (T_RM_, CD4^+^, and CD69^+^), CD4^+^ memory T cells (Memory T, CD4^+^, and CD44^+^), regulatory T cells (T_Reg_, CD4^+^, and CD25^+^), CD8^+^ naive T cells (CD8^+^ and CD62L^+^), γδ T cells (T_γδ_, CD3^+^, and TCR_γδ_^+^), and natural killer T cells (NKT, CD3^+^, and NK1.1^+^). For mock-infected mice, estradiol treatment was performed, and mice were inoculated vaginally with 10 µL sterile PBS in place of bacterial infection.

### Statistical analyses

All experiments were completed in at least duplicate unless otherwise indicated, with results combined prior to analyses. Experimental sample size (*n*) and exact tests used are indicated in figure legends. Data normality was tested using the D’Agostino-Pearson normality test. Data were analyzed according to the caption under each corresponding figure. For all experiments, a *P*-value or *q*-value of <0.05 was considered statistically significant. Statistical analyses were performed using GraphPad Prism v9.5.1, R v4.3.3, or Python v3.9.2.

## Data Availability

Raw sequencing files can be found in the NCBI Sequence Read Archive (SRA) and associated BioProject, accession PRJNA1394477. Code for data analysis can be found in the Patras Lab Github: https://github.com/PatrasLab/ T2D_GBS_mouse_vaginal_colonization_manuscript.

## SUPPLEMENTAL MATERIAL

Supplemental Material msphere.00027-26-s0001.pdf Figures S1-S5 and captions for Tables S1 and S2.

Table S1 msphere.00027-26-s0002.xlsx Supplemental data for 16S rRNA gene amplicon sequencing.

Table S2 msphere.00027-26-s0003.xlsx Source data and Spearman correlations for metabolic markers, GBS burdens, cytokines, panel of flow cytometry markers, and immune cell populations.

ASM does not own the copyrights to Supplemental Material that may be linked to, or accessed through, an article. The authors have granted ASM a non-exclusive, world-wide license to publish the Supplemental Material files. Please contact the corresponding author directly for reuse.
