# Supplementary material for: Type 2 diabetes mellitus exacerbates vaginal group B Streptococcus colonization via impaired mucosal cytokine response
Source: mSphere. 2026 Jul 10;11(7):e00027-26. doi: 10.1128/msphere.00027-26 (PMC13410995; doi:10.1128/msphere.00027-26)
Supplement: Supplemental Material — Figures S1-S5 and captions for Tables S1 and S2. [file msphere.00027-26-s0001.pdf]

SUPPLEMENTAL MATERIALS

Type 2 diabetes mellitus exacerbates vaginal group B Streptococcus colonization via impaired mucosal cytokine response

Clare M. Robertson, Vicki Mercado-Evans, Addison B. Larson, Holly Branthoover, Samantha Ottinger, Marlyd E. Mejia, Zainab A. Hameed, Lindsey A. Gonzalez, Camille Serchejian, Libbie Ogilvie, Jacob J. Zulk, Kathryn A. Patras

Contents:

Supplemental Figures 1-5

Captions for Supplemental Tables 1-2

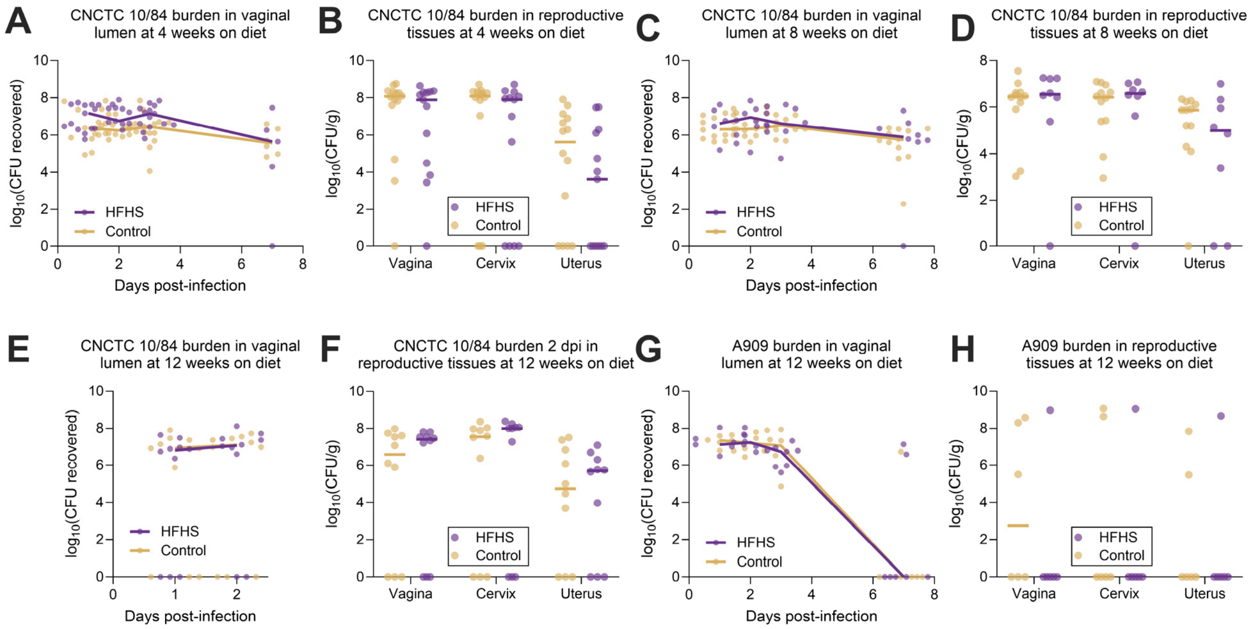

Figure S1: GBS burdens in mice with shorter diet regimens and alternative GBS strains. Mice were subjected to a 4-, 8-, or 12-week diet course and GBS burdens were quantified from post-infection vaginal swabs or post-infection homogenized reproductive tissues. GBS CNCTC 10/84 burdens in vaginal swabs (**A**) or tissues 7 days post-infection (dpi) (**B**) after a 4-week diet course. GBS CNCTC 10/84 burdens in vaginal swabs (**C**) or tissues 7 dpi (**D**) after an 8-week diet course. GBS CNCTC 10/84 burdens in vaginal swabs (**E**) or tissues 2 dpi (**F**) after a 12-week diet course. GBS A909 burdens in vaginal swabs (**G**) or tissues 7 dpi (**H**) after a 12-week diet course. *n*=13-14 (A-B), *n*=8-12(C-D), *n*=10(F-F), *n*=6-7(G-H). Data represent 2 independent experiments (A-H). Points indicate individual samples, and lines or curves indicate medians. Data were analyzed by two-way ANOVA with Benjamini, Krieger and Yekutieli correction and false discovery rate (FDR) set at 5% (A-H) and no significant differences were detected. Supplemental to Figure 1.

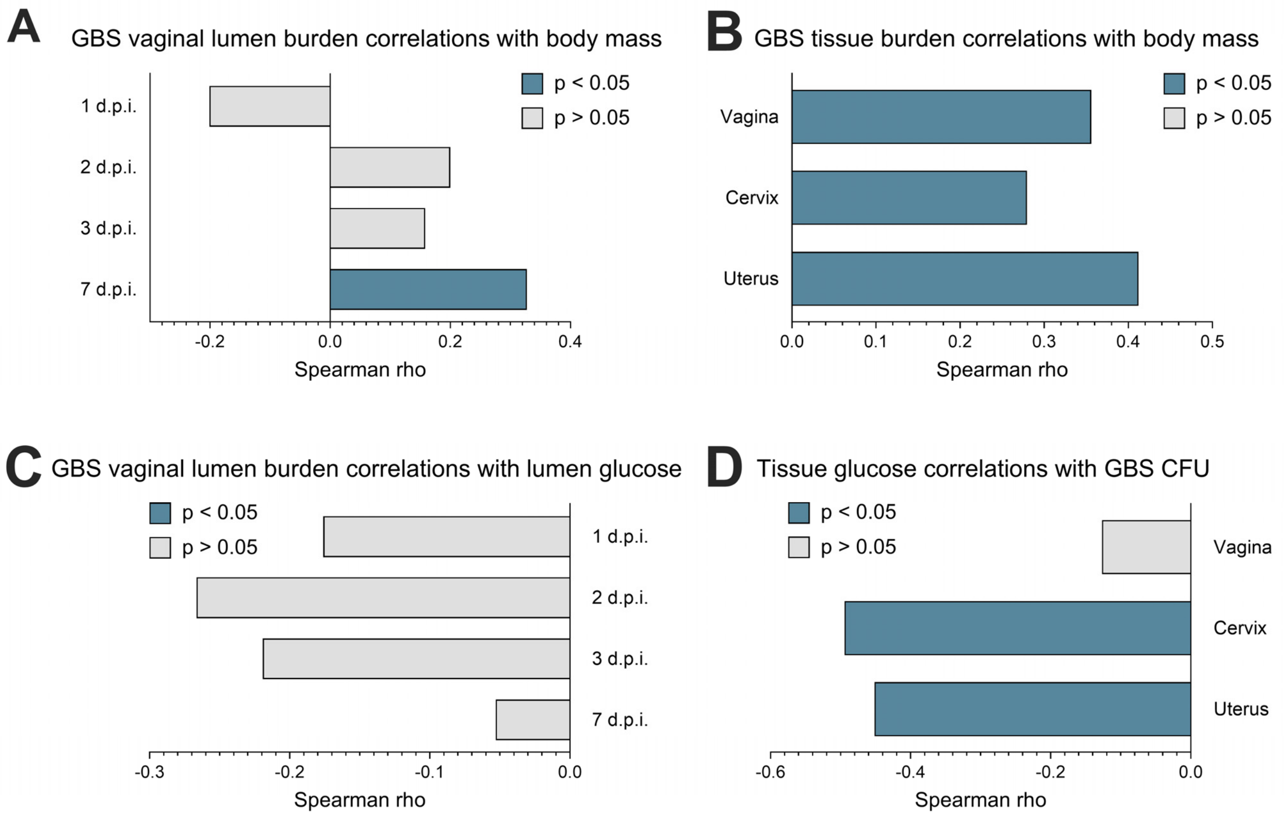

Figure S2: Body mass and reproductive tract glucose correlations with GBS burdens. Spearman correlations of body mass with GBS vaginal lumen CFU (**A**) or GBS tissue burdens at 7 days post-inoculation (dpi) (**B**). **C**) Spearman correlations of baseline (12-week diet course) vaginal lumen glucose with GBS vaginal lumen CFU post-inoculation. **D**) Spearman correlations of 7dpi tissue glucose levels with their respective GBS CFU 7dpi. *n*=16-28. Data represent 4 (A-B, D) and 3 (C) independent experiments. Data were analyzed by Spearman correlation with Benjamini-Hochberg FDR correction. Supplemental to Figure 1.

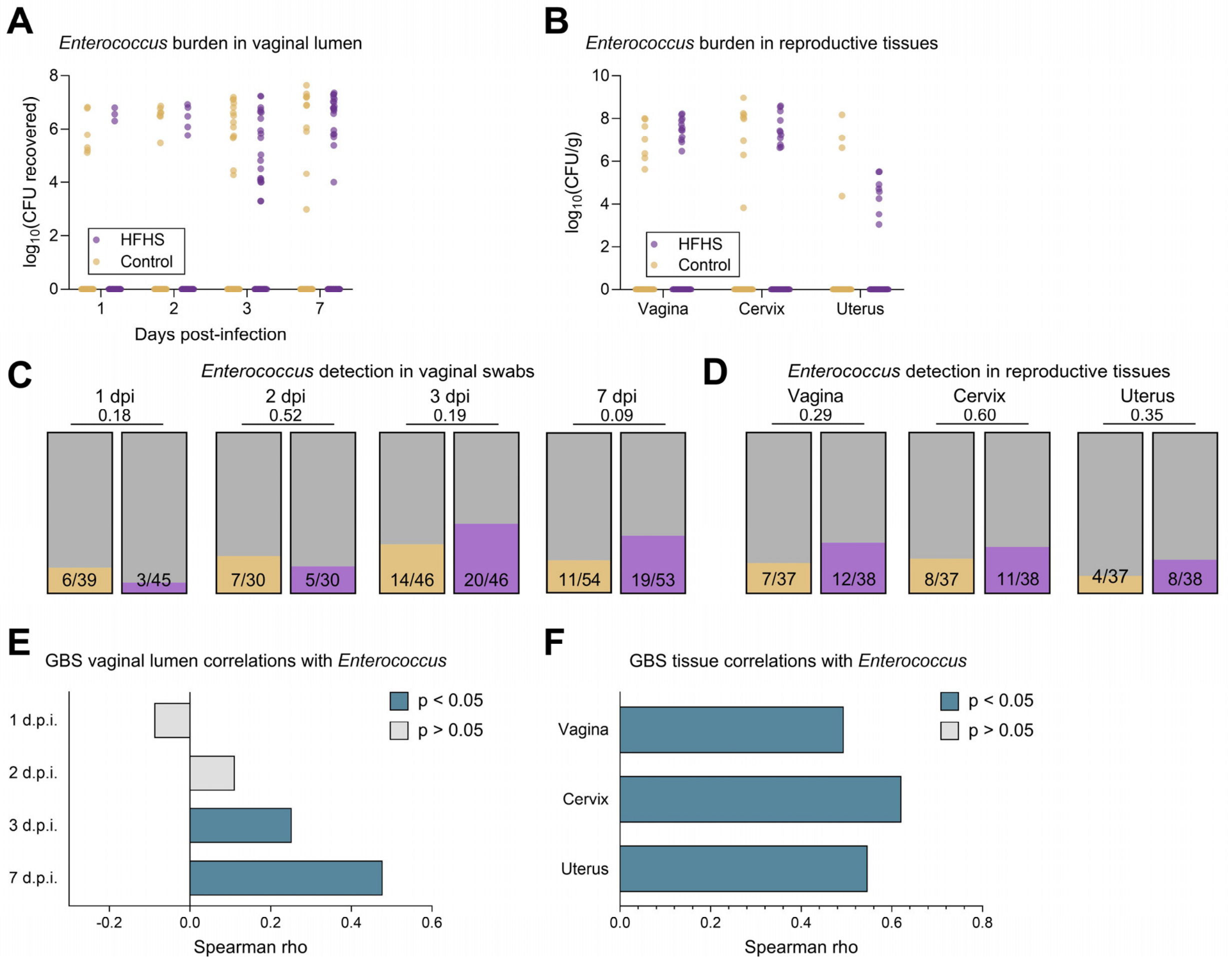

Figure S3: *Enterococcus* in vaginal lumen and reproductive tract tissues. **A**) *Enterococcus* burdens from post-infection vaginal swabs. **B**) *Enterococcus* burdens from post-infection homogenized reproductive tissues at 7 days post-infection (dpi). **C**) Proportions of mice in each group (control: yellow, HFHS: purple) with *Enterococcus* detected in vaginal swab samples. **D**) Proportions of mice in each group with *Enterococcus* detected in reproductive tract tissues at 7 dpi. Spearman correlations between GBS CFU and *Enterococcus* CFU in vaginal swabs at all timepoints (**E**) and in reproductive tissues at 7 dpi (**F**). *n*=30-54 (A,C,E), *n*=37-38 (B,D,F). Data represent 4 independent experiments. Points indicate individual samples, and lines indicate medians. Data were analyzed by two-way ANOVA with Benjamini, Krieger and Yekutieli correction and false discovery rate (FDR) set at 5% (A-B) or Fisher's Exact test (C-D) and no significant differences were detected. Data were analyzed by Spearman correlation with Benjamini-Hochberg FDR correction (E-F). Supplemental to Figure 2.

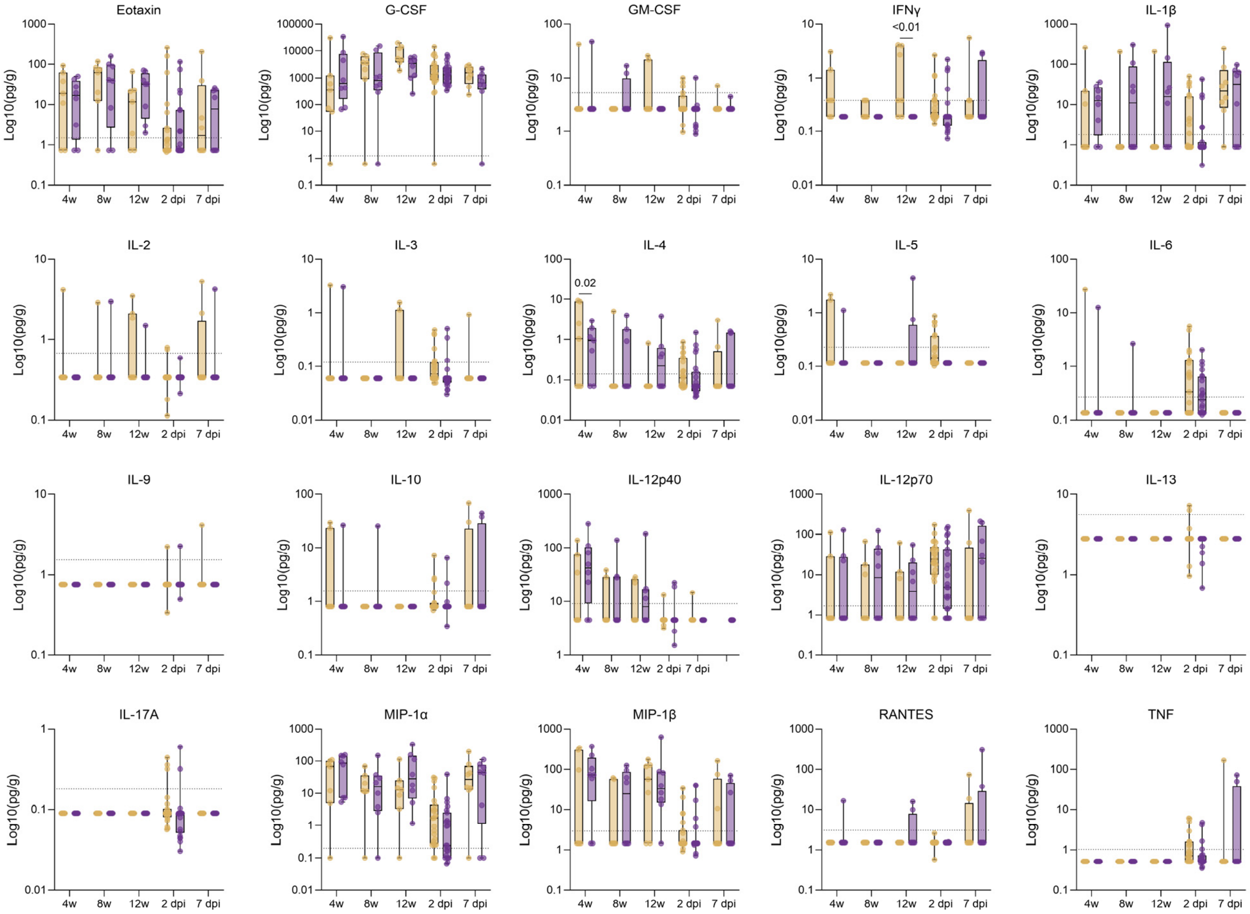

Figure S4: Vaginal cytokine concentrations at all timepoints. Cytokine concentrations of vaginal lavage fluid taken pre- and post-infection, normalized to total protein. Data represent 1 (pre-infection and 7 dpi timepoints) or 3 (2 dpi timepoint) independent experiments. Horizontal dotted lines represent lower limit of detection (LLOD). Concentrations that were below the LLOD were extrapolated from standard curves where possible. Where extrapolation was not possible, the values were imputed as half the LLOD. Data were analyzed by two-tailed Mann−Whitney *U* test between diet groups at matched timepoints and all comparisons were not significant, $p>0.05$. Supplemental to Figure 3.

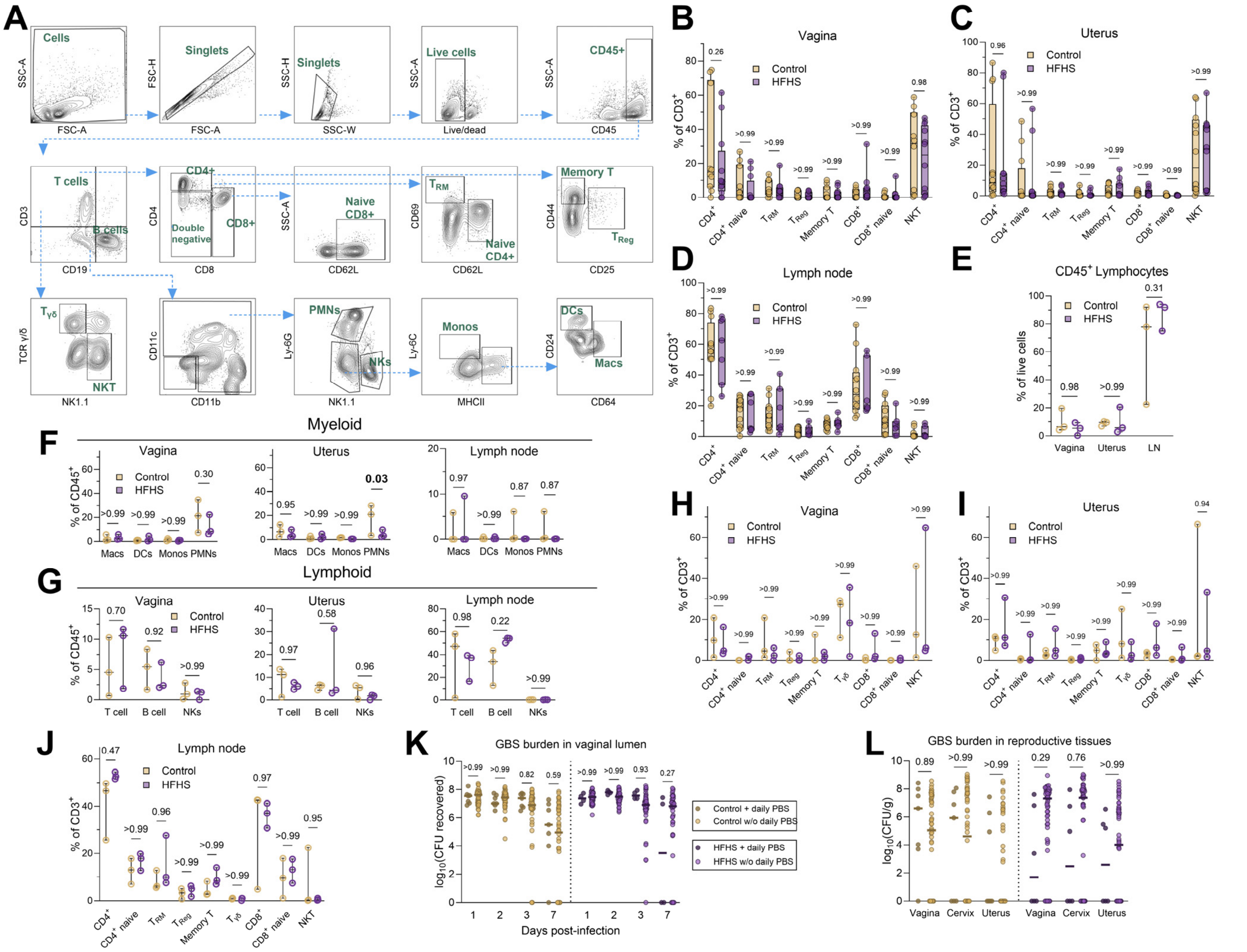

Figure S5: Immune cell profiling and impact of carrier treatment on GBS burdens. **A**) Gating strategy for immune cell profiling via flow cytometry. Vagina (**B**), uterus (**C**), and ileal lymph node (**D**) T cell subsets in GBS-infected mice at 2dpi. Immune cell proportions in mock-infected mice at 2 days post-treatment presented as total CD45$^+$ cells out of live cells (**E**), myeloid lineages (**F**), lymphoid lineages (**G**), and T cell subsets in vagina (**H**), uterus (**I**), and ileal lymph node (**J**). Cell populations were defined as follows: lymphocytes (CD45$^+$), T cells (CD3$^+$), B cells (CD19$^+$), natural killer cells (NKs, NK1.1$^+$, CD11b^{var}), dendritic cells (DCs, CD11b^{var}, CD11c^{var}, MHC-II$^+$, CD24$^+$), macrophages (Macs, CD11b^{var}, CD11c^{var}, MHC-II$^+$, CD64$^+$), monocytes (Monos, CD11b^{var}, CD11c^{var}, MHC-II$^-$, Ly6C$^+$), neutrophils (PMNs, CD11b^{var}, Ly6G$^+$), CD4 T cells (CD3$^+$, CD4$^+$), CD8 T cells (CD3$^+$, CD8$^+$), CD4$^+$ naïve T cells (CD4$^+$, CD62L$^+$), resident memory T cells (T_{RM}, CD4$^+$, CD69$^+$), CD4$^+$ memory T cells (Memory T, CD4$^+$, CD44$^+$), regulatory T cells (T_{Reg}, CD4$^+$, CD25$^+$), CD8+ naïve T cells (CD8$^+$, CD62L$^+$), γδ T cells ($T_{\gamma\delta}$, CD3$^+$, TCR$_{\gamma\delta}^+$), and natural killer T cells (NKT, CD3$^+$, NK1.1$^+$). As a mock treatment, mice were dosed daily with the rIL-1α diluent/carrier (PBS). GBS burdens were quantified from post-infection vaginal swabs or post-infection homogenized reproductive tissues. GBS burdens in vaginal swabs (**K**) or tissues 7 days post-infection (dpi) (**L**) are compared between mock-treated (daily PBS) mice and mice from previous 12-week diet GBS infection experiments (w/o daily PBS). Immune cell data represent 4 independent experiments. *n*=10-12 per group (B-D), *n*=3 per group (E-J), or *n*= 4-57 per group (K-L). Plots show all points, representing individual samples, lines indicate medians, and whiskers extend from minimum to maximum. Data were analyzed by two-way ANOVA with Šidák correction for multiple comparisons (B-L). Supplemental to Figures 3 and 4.

Supplemental Table 1. Supplemental data for 16S rRNA gene amplicon sequencing. ASV counts with their associated taxonomy and consensus sequence, ASVs removed during decontamination, and results of differential abundance and presence analyses. Data represent 2 independent experiments, n=8-16 per timepoint. Supplemental to Figure 2.

Supplemental Table 2. Source data and Spearman correlations for metabolic markers, GBS burdens, cytokines, and immune cell populations. Spearman correlations between metabolic markers and cytokines at indicated timepoints, Spearman correlations between GBS CFU and cytokines at indicated timepoints, source data for GBS CFU and cytokine (pg/g of total protein) assays, panel of markers for flow cytometry, and immune cell populations (as % of live cells, $CD45^+$, or parent). Supplemental to Figures 3, 4, S4, and S5.
